# Donor Blood Tests do Not Predict Pancreas Graft Survival After Simultaneous Pancreas Kidney Transplantation; a National Cohort Study

**DOI:** 10.3389/ti.2024.12864

**Published:** 2024-05-20

**Authors:** Ning Xuan Ho, Samuel J. Tingle, Abdullah K. Malik, Emily R. Thompson, Georgios Kourounis, Aimen Amer, Sanjay Pandanaboyana, Colin Wilson, Steve White

**Affiliations:** ^1^ National Institute for Health Research Blood and Transplant Research Unit (NIHR BTRU) in Organ Donation and Transplantation, Institute of Transplantation, Freeman Hospital, Newcastle upon Tyne, United Kingdom; ^2^ Translational and Clinical Research Institute, Newcastle University, Newcastle upon Tyne, United Kingdom

**Keywords:** pancreas transplantation, SPK transplantation, registry study, donor blood tests, graft surival, organ utilisation

## Abstract

Simultaneous pancreas-kidney (SPK) transplantation improves quality of life and limits progression of diabetic complications. There is reluctance to accept pancreata from donors with abnormal blood tests, due to concern of inferior outcomes. We investigated whether donor amylase and liver blood tests (markers of visceral ischaemic injury) predict pancreas graft outcome using the UK Transplant Registry (2016-2021). 857 SPK recipients were included (619 following brainstem death, 238 following circulatory death). Peak donor amylase ranged from 8 to 3300 U/L (median = 70), and this had no impact on pancreas graft survival when adjusting for multiple confounders (aHR = 0.944, 95% CI = 0.754–1.81). Peak alanine transaminases also did not influence pancreas graft survival in multivariable models (aHR = 0.967, 95% CI = 0.848–1.102). Restricted cubic splines were used to assess associations between donor blood tests and pancreas graft survival without assuming linear relationships; these confirmed neither amylase, nor transaminases, significantly impact pancreas transplant outcome. This is the largest, most statistically robust study evaluating donor blood tests and transplant outcome. Provided other factors are acceptable, pancreata from donors with mild or moderately raised amylase and transaminases can be accepted with confidence. The use of pancreas grafts from such donors is therefore a safe, immediate, and simple approach to expand the donor pool to reach increasing demands.

## Graphical Abstract

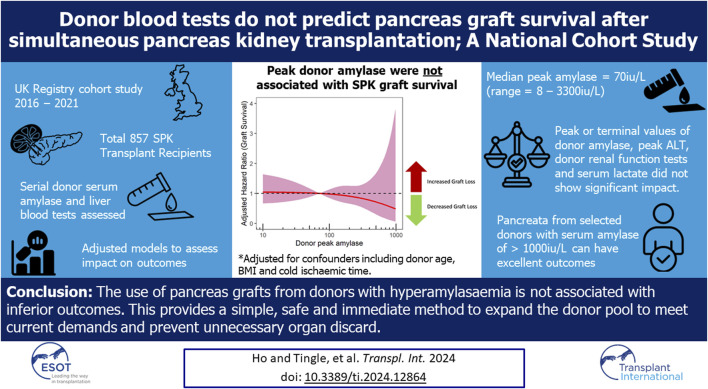

## Introduction

Diabetes Mellitus (DM) is a growing pandemic [1–3] associated with increased risks of developing life-limiting systemic complications. Diabetic patients may experience reduced quality of life and incur high healthcare-associated costs, particularly in patients with poorly controlled disease. Pancreas transplantation significantly improves the quality of life of patients, and can limit the progression of serious medical comorbidities [4–7].

The number of patients on the UK waiting list for pancreas transplantation is at an all-time high, and waiting time has worsened following the COVID-19 pandemic [8]. As of March 2023, 265 patients are actively waiting for a pancreas graft in the United Kingdom, representing a 27% increase from before the pandemic [9]. Taken together, there is a need to optimise decision-making surrounding organ utilisation and expand the donor pool to match the current demands for pancreas transplantation. It is essential to understand factors that predict transplant outcomes. It is equally important to identify factors that do not lead to poor outcomes, preventing the unwarranted rejection of donor organs based on these factors.

Initial screening of donors includes various blood tests, such as serum amylase and liver blood tests. Hyperamylaseamia (defined as serum amylase levels greater than 110 UI/L) can be seen in up to 40% of donors, and a markedly elevated serum amylase (more than three times the upper limit of normal) is generally considered to represent pancreatitis [10]. However, this blood test has low specificity, and can be raised due to a variety of aetiologies [11, 12].

Serum liver blood tests (LBTs) are markers of acute hepatocellular or cholangiocyte injury. The embryological development of the pancreas is closely related to the formation of foregut and midgut structures. The pancreas shares the same vascular supply with other foregut/midgut structures (including liver), receiving blood from both coeliac trunk and superior mesenteric artery. Therefore, markers of acute hypoxic injury to the liver could be a surrogate for hypoxic injury to the pancreas [13, 14].

This study aims to ascertain whether donor amylase and LBTs predict pancreas graft survival in patients undergoing SPK transplantation.

## Materials and Methods

Data on adult simultaneous pancreas and kidney (SPK) transplants was retrieved from the UK Transplant registry, maintained by the National Health Service Blood and Transplant (NHSBT). Adult recipients (>16 years) from all 8 UK pancreas transplant centres, transplanted between January 2016 and December 2021, were included. These dates were chosen because, before January 2016, serial donor amylase and serial LBTs were not recorded. Recipients of grafts donated following circulatory or brain stem death [donation following brain stem death (DBD)/donation following circulatory death (DCD)] were included.

Data were provided in an anonymized form (patient identifiable information and transplant unit not provided) as per NHSBT approvals, and individual ethical or institutional review board approval was not required for this project. This project was approved by the pancreas advisory board.

Data were extracted from NHSBT in August 2023. Data were cleaned, and values that were deemed impossible were removed. Our primary aim was to compare the impact of donor serum amylase on 3-year pancreas graft survival. Secondary analyses compared the impact of donor alanine transaminase (ALT), aspartate transaminase (AST), alkaline phosphatase (ALT) and bilirubin, as well as renal blood tests and lactate, on 3-year pancreas graft survival.

Graft loss was defined as retransplantation, pancreatectomy or return to insulin therapy due to graft failure and was analysed as time-to-event, death censored, and measured until July 2023 (the common closure date of the study).

### Statistical Analysis

Missing data is summarised in Supplementary Table S1. Missing data were dealt with by multiple imputation using the fully conditional specification technique applied to generate 5 imputed datasets. Due to significant right skew, peak amylase, LBT, renal function test and serum lactate values were log transformed prior to performing multiple imputation. These imputed datasets were used for all multivariable models.

Our approach for constructing multivariable models matched that described previously [13]. When entering LBT values as predictors in the following models they were kept as continuous variables, rather than splitting into arbitrary categories; this approach improves power and is best practice. The blood tests were kept as continuous variables, which is superior to creating arbitrary categories [15–17]. To combat issues with skew, all blood test values were entered into models as log2 (blood test value).

Cox proportional hazards method was used to build multivariable graft survival models. Donor, graft, recipient and operative factors available from NHSBT registry were initially screened. Variables were selected based on clinical experience, if they had previously been reported to affect graft survival, or if they were significantly correlated with donor amylase and LBTs. Table 3 lists all considered variables. Automatic variable selection techniques (such as backwards stepwise selection) were avoided as these are recommended against in small datasets [18].

As there was significant correlation between each of the blood tests, there would be significant issues with multi-collinearity if they were entered into the same model. Therefore, separate multivariable models were built for donor amylase and each individual LBT, renal function test and serum lactate values. Results of these models are displayed as adjusted hazard ratios (aHR) with 95% confidence intervals. Interaction terms where introduced into these models to assess whether the impact of donor blood tests on pancreas graft survival differed in older donors or those with prolonged CIT.

Finally, we repeated our main cox regression models for graft survival, using a restricted cubic spline approach (3 knots located at 10/50/90th percentiles) to assess the impact of donor serum amylase and LBTs on outcome without assuming linear relationships [19].

For all tests performed *p* < 0.05 was deemed significant. Analyses were performed in SPSS™ version 26 (IBM Corp, Armonk, New York, United States) or R (R Foundation for Statistical Computing, Vienna, Austria). The latter was used to generate all figures.

## Results

857 adult recipients of deceased donor pancreas (619 DBD and 238 DCDs) were included, with median follow up of 37.5 months. Median donor age was 34 (interquartile range 24–46). Cohort demographics are included in Table 1, with further details in Supplementary Table S1.

**TABLE 1:** Summary of Cohort Demographics (*N* = 857).

	DBD (N = 619)	DCD (N = 238)	Overall (N = 857)
Recipient Age			
Median [Min, Max]	42.0 [21.0, 64.0]	42.0 [20.0, 61.0]	42.0 [20.0, 64.0]
Recipient Sex			
Female	267 (43.1%)	92 (38.7%)	359 (41.9%)
Male	352 (56.9%)	146 (61.3%)	498 (58.1%)
Recipient Ethnicity			
White	515 (83.2%)	205 (86.1%)	720 (84.0%)
Non-White	97 (15.7%)	32 (13.4%)	129 (15.1%)
Recipient BMI			
Median [Min, Max]	24.6 [17.7, 36.5]	25.1 [18.4, 36.9]	24.8 [17.7, 36.9]
Type of Recipient Diabetes			
Type 1 Diabetes Mellitus	488 (78.8%)	179 (75.2%)	667 (77.8%)
Type 2 Diabetes Mellitus	22 (3.6%)	10 (4.2%)	32 (3.7%)
Donor Sex			
Female	316 (51.1%)	96 (40.3%)	412 (48.1%)
Male	303 (49.0%)	142 (59.7%)	445 (51.9%)
Donor Age			
Median [Min, Max]	35.0 [10.0, 63.0]	29.0 [4.00, 54.0]	34.0 [4.00, 63.0]
Donor BMI			
Median [Min, Max]	23.4 [14.5, 38.4]	22.6 [11.3, 36.2]	23.1 [11.3, 38.4]
Donor Ethnicity			
White	554 (89.5%)	216 (90.8%)	770 (89.8%)
Non-White	53 (8.6%)	21 (8.8%)	74 (8.6%)
Donor Cause of Death			
Hypoxic Brain Injury	198 (32.0%)	111 (46.6%)	309 (36.1%)
Intrcranial Haemorrhage	284 (45.9%)	61 (25.6%)	345 (40.3%)
Intrcranial Thrombosis	27 (4.4%)	9 (3.8%)	36 (4.2%)
Trauma	30 (4.8%)	25 (10.5%)	55 (6.4%)
Other	55 (8.9%)	17 (7.1%)	72 (8.4%)
Cold Ischaemic Time (minutes)			
Median [Min, Max]	647 [223, 1,320]	611 [339, 1,060]	634 [223, 1,320]
3-year Graft failure			
No	547 (88.4%)	214 (89.9%)	761 (88.8%)
Yes	68 (11.0%)	21 (8.8%)	89 (10.4%)

DBD, donation following brainstem death; DCD, donation following circulatory death.

### Summary of Donor Serum Amylase and Liver Blood Tests

**FIGURE 1:**
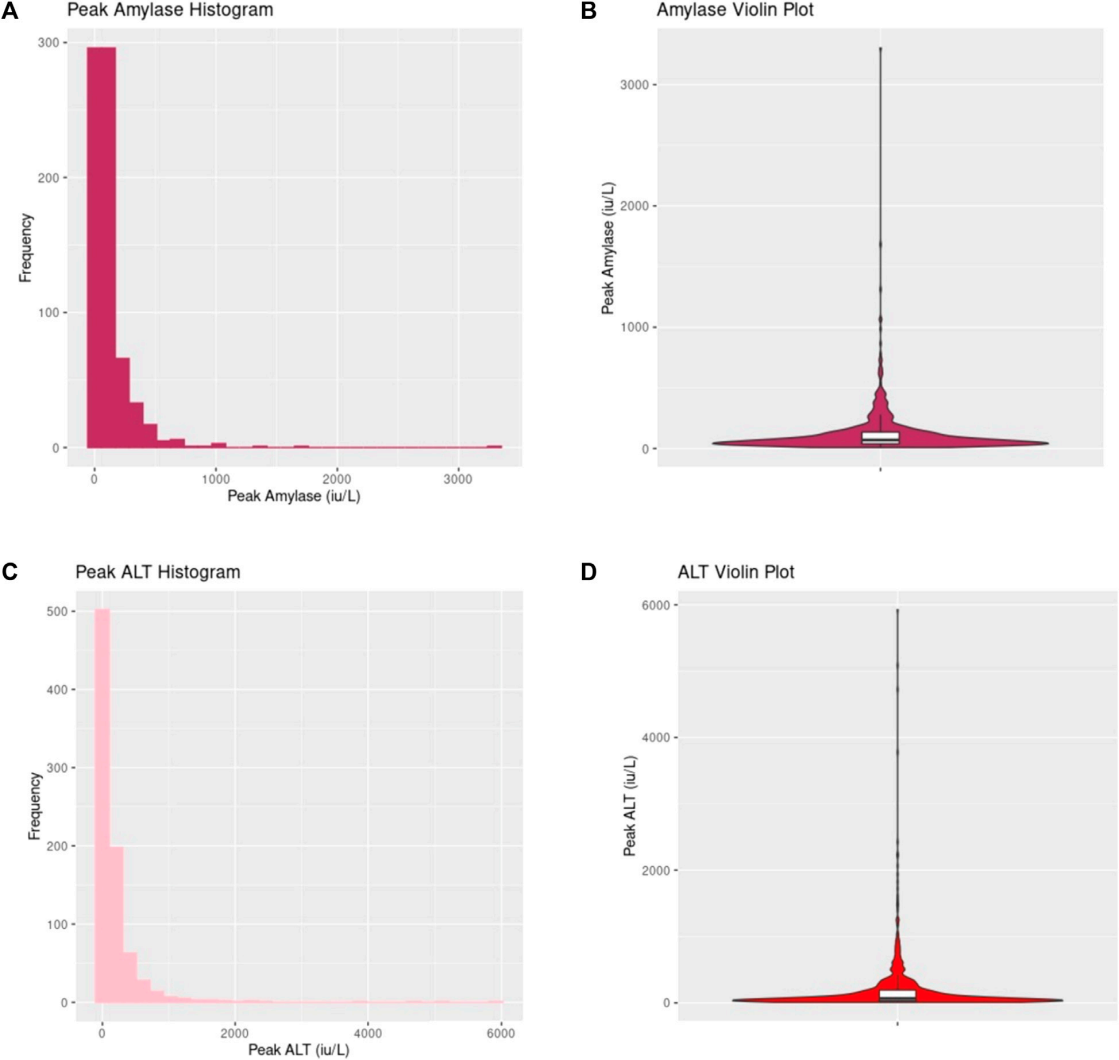
Donor peak amylase and peak alanine transaminase (ALT) distribution. **(A,B)** demonstrates values of peak amylase across the entire cohort displayed in histogram and violin plot respectively. **(C,D)** shows values of peak ALT across the entire cohort displayed in histogram and violin plot respectively.

Table 2 provides a summary of donor amylase and liver blood tests across the cohort (see Supplementary Material S2 for further details). Peak Amylase and ALT values are graphically displayed in Figure 1. A wide range of peak donor amylase were identified in our study. 465 donors had a peak amylase of <100 iu/L, 257 donors had a peak amylase of between 100 iu/L and 1000 iu/L, and five donors had peak amylase >1000 iu/L (130 were missing a value for peak amylase). Of all donors, a total of 197 had an amylase value of >130 iu/L (the P-PASS cut-off) [20].

**TABLE 2:** Summary of Peak Donor Serum Amylase and Liver Blood Tests.

	DBD (N = 619)	DCD (N = 238)	Overall (N = 857)
Amylase			
Median [Min, Max]	70 [8, 3,300]	69 [10, 1,310]	70.0 [8, 3,300]
ALT			
Median [Min, Max]	59 [8, 5,090]	89 [9, 5,930]	67.0 [8, 5,930]
AST			
Median [Min, Max]	65 [0, 2040]	94.0 [10, 7,910]	72.0 [0, 7,910]
ALP			
Median [Min, Max]	85 [31, 721]	90.0 [35, 541]	86.0 [31, 721]
Bilirubin			
Median [Min, Max]	12 [3, 124]	11.5 [3, 65]	12.0 [3, 124]

DBD, donation following brainstem death; DCD, donation following circulatory death; ALT, alanine transaminase; AST, aspartate transaminase; ALP, alkaline phosphatase.

Supplementary Figure S1 provides a graphical display of peak donor AST, ALP and bilirubin values. There were no significant differences in the blood tests between DBD and DCD donors (Table 2).

### Impact of Amylase and Liver Blood Tests on Pancreas Graft Survival

Table 3 displays the multivariable cox regression model for 3-year pancreas graft survival. Peak donor amylase, peak transaminases (ALT and AST), peak ALP, and peak bilirubin did not predict pancreas graft survival, even when adjusting for a range of factors (Table 3).

**TABLE 3:** 3-Year Graft Survival Cox regression using pooled data on peak donor amylase and liver blood tests from imputed datasets.

	Adjusted HR (95% CI)	*p*-value
Blood Tests		
Amylase (Peak)	0.944 (0.754–1.181)	0.602
ALT (Peak)	0.967 (0.848–1.102)	0.616
AST (Peak)	0.908 (0.771–1.070)	0.247
ALP (Peak)	0.865 (0.594–1.261)	0.451
Bilirubin (Peak)	1.229 (0.930–1.624)	0.148
Cold Ischaemic Time (hours)	1.338 (0.611–2.930)	0.467
Donor Age (years)	1.009 (0.992–1.026)	0.322
Donor Type	0.731 (0.430–1.243)	0.247
Donor BMI	1.078 (1.015–1.144)	0.014
Transplant Year	0.948 (0.820–1.096)	0.472
Recipient Age (years)	0.960 (0.935–0.986)	0.003
Recipient BMI	0.992 (0.918–1.073)	0.842

For blood tests, logs were taken before inclusion in this model, due to all blood tests results being right-skewed. The effect estimates relate to a unit increase in log2 (blood tests value). Results from the various LBTs (ALT, AST, ALP, and bilirubin) could not be included in a single model because of multicollinearity; therefore, multivariable results for each LBT are from a separate multivariable model. Multivariable results for variables other than LBTs are from the model including peak Amylase.

ALT, alanine transaminase; AST, aspartate transaminase; ALP, alkaline phosphatase; CI, confidence interval; LBT, liver blood test; HR, hazard ratio; DBD, donation following brainstem death; DCD, donation following circulatory death.

The impact of blood tests on outcome was then assessed separately in DBD and DCD cohorts. Repeating the model in Table 3 in the DBD cohort, confirmed that donor amylase did not predict pancreas graft survival in this group (aHR = 0.965, 0.760–1.227, *p* = 0.768). For DCD graft recipients, a further multivariable model was created, with the addition of normothermic regional perfusion (NRP) as a confounder; again, this confirmed no impact of donor amylase on pancreas graft survival (aHR = 0.984, 0.609–1.590, *p* = 0.948). Similar analyses found no impact of peak donor liver blood tests in either the DBD or DCD subgroup.

We have also performed a multivariable analysis on those with amylase values greater than 130 (the cut-off used in the P-PASS score) [20], adjusting for all of the factors in Table 3. Pancreases from donors with peak amylase >130 were not at higher risk of graft loss compared with those with amylase ≤130 (aHR = 0.730, 95% CI 0.460–1.733, *p* = 0.730). This is a sensitivity analysis only, as using arbitrary cut-offs for continuous variables significantly reduces the power of analyses.

Donor amylase and transaminases may have a greater impact in older donors and pancreases with prolonged cold ischaemic time. This hypothesis was tested by the addition of interaction terms to the model shown in Table 3. There was no evidence that the impact of donor peak amylase or peak ALT on pancreas graft survival differed based on donor age (interaction *p* = 0.340 & *p* = 0.890 respectively), or prolonged cold ischaemic time (interaction *p* = 0.699 & *p* = 0.924 respectively).

**FIGURE 2:**
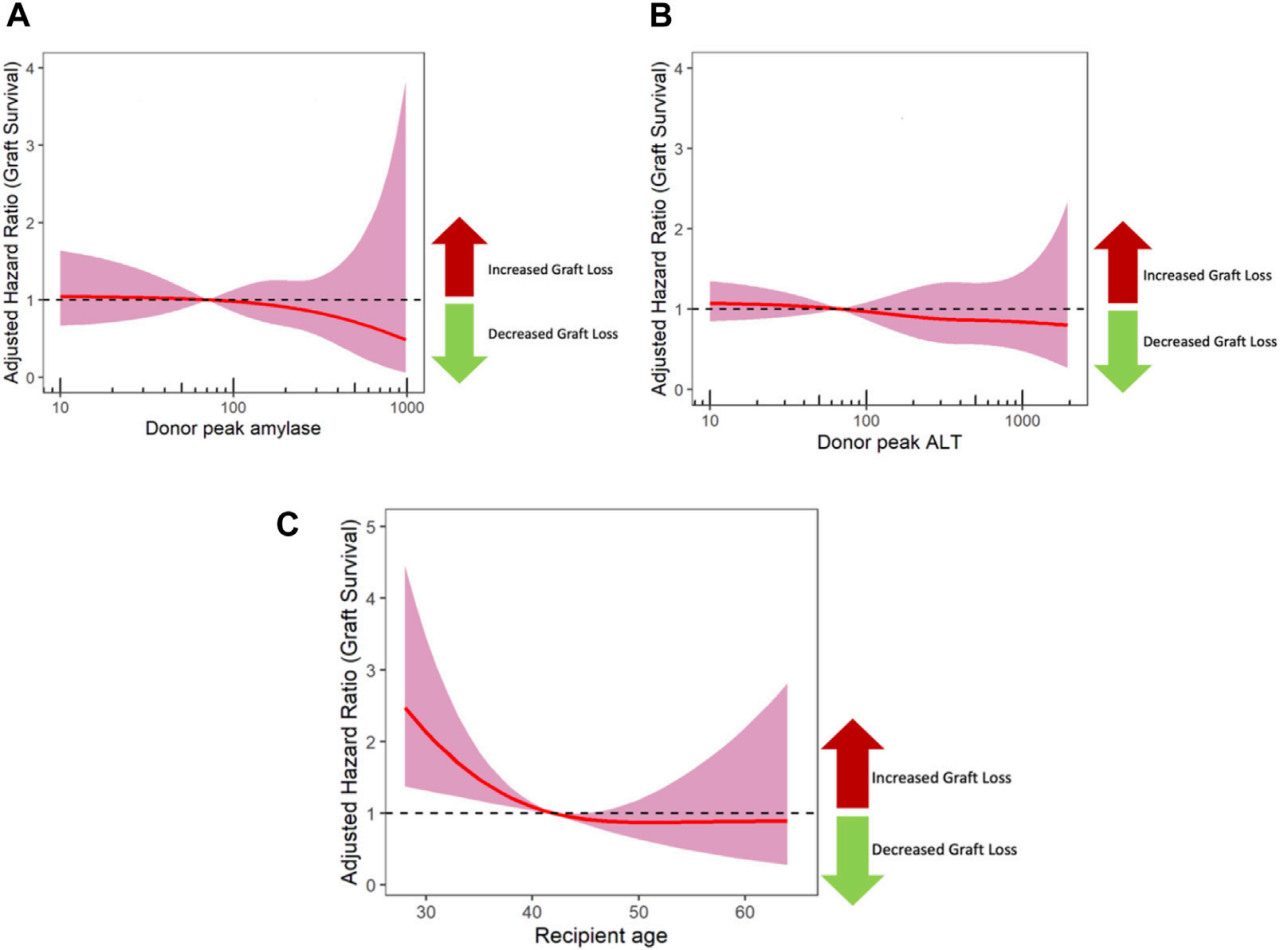
The impact of peak donor Amylase **(A)** and ALT **(B)** on graft survival using cox regression models with restricted cubic splines. The shaded area represents the 95% confidence interval, and a dashed line at 1 represents no impact on outcome. For comparison, a separate model was performed for recipient age **(C)**, which showed that younger recipients have worse outcome. ALT, alanine transaminase.

The relationship between peak amylase/LBT values and graft survival was also modelled using restricted cubic splines (Figures 2A, B). This avoids assumptions about the nature of the relationship between peak blood test values and outcome, whilst also adjusting for all the confounders listed in Table 3. As shown in Figures 2A, B, this confirms no impact of peak amylase or peak ALT on outcome. By way of counter example, a restricted cubic spline analysis was also performed for recipient age which is a known prognostic factor; this showed that younger recipients have worse outcome (Figure 2C).

It may be argued that the terminal value (the value closest to donation) is more predictive of outcome. As serum amylase and LBT levels closest to donation (rather than peak values) may represent the cumulative effect of ischaemic injury during donation, we built further models using terminal values in an identical fashion to Table 3. This is shown in Table 4, where terminal values of amylase, LBTs, renal function tests and serum lactate were not significant in outcomes.

**TABLE 4:** Sensitivity Analyses with terminal values of amylase and liver blood tests.

	Adjusted HR (95% CI)	*p*-value
Blood Tests		
Amylase (Terminal)	0.979 (0.776–1.236)	0.857
ALT (Terminal)	0.965 (0.828–1.124)	0.646
AST (Terminal)	0.895 (0.669–1.198)	0.430
ALP (Terminal)	1.011 (0.713–1.434)	0.950
Bilirubin (Terminal)	1.067 (0.798–1.428)	0.661
Cold Ischaemic Time (hours)	1.348 (0.614–2.957)	0.457
Donor Age (years)	1.009 (0.992–1.026)	0.297
Donor Type	0.736 (0.433–1.251)	0.258
Donor BMI	1.077 (1.015–1.144)	0.015
Transplant Year	0.949 (0.821–1.098)	0.484
Recipient Age (years)	0.960 (0.935–0.986)	0.003
Recipient BMI	0.993 (0.919–1.073)	0.857

For blood tests, logs were taken before inclusion in this model, due to all blood tests results being right-skewed. The effect estimates relate to a unit increase in log2 (blood tests value). Results from the various LBTs (ALT, AST, ALP, and bilirubin) could not be included in a single model because of multicollinearity; therefore, multivariable results for each LBT are from a separate multivariable. Multivariable results for variables other than LBTs are from the model including peak Amylase.

ALT, alanine transaminase; AST, aspartate transaminase; ALP, alkaline phosphatase; CI, confidence interval; LBT, liver blood test; HR, hazard ratio; DBD, donation following brainstem death; DCD, donation following circulatory death.

**FIGURE 3:**
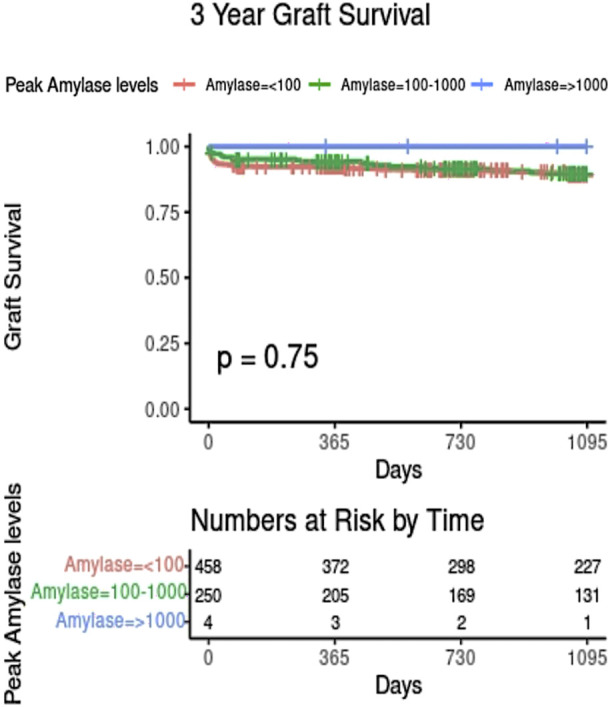
Kaplan-Meier plot showing graft survival based on donor peak amylase level. Only those with complete amylase and graft survival data are shown.

There may be specific concern where donor amylase values are extremely elevated (>1000 iu/L, 10 times the upper limit of normal). Follow-up data was available for 4 pancreas transplants which used grafts from donors with peak amylase over 1,000; all of these were functioning at last follow-up (Figure 3).

Sensitivity analyses were performed where raw amylase and LBT values (rather than log-transformed values) were entered into the cox regression model. Again, peak donor amylase and LBTs did not show significant impact in recipient outcomes.

We also assessed the impact of donor renal function tests and lactate, as the function of the transplanted kidney can impact pancreas graft function. Donor HbA1c was not recorded for more than 90% of the donors and therefore could not be assessed in this study. Donor peak creatinine, peak urea, peak estimated glomerular filtration rate (eGFR), and serum lactate did not predict pancreas graft survival (Supplementary Table S3). None of the examined blood tests predicted kidney graft survival in multivariable models. However, kidney graft survival may be better assessed in a study dedicated to kidney grafts, with much larger cohorts of kidney transplants alone.

## Discussion

This large, statistically robust cohort study (619 DBD and 238 DCDs) has found no association between donor amylase and pancreas graft survival in SPK transplantation, on adjusted analyses. Although there was no evidence of an impact on outcome at any donor amylase level, relatively few pancreases were transplanted from donors with extreme increases in amylase (>1,000). Therefore, the impact of extreme elevations in amylase remain uncertain and such donor should be assessed on a case-by-case basis. It is reassuring that all four pancreases transplanted from donors with amylase >1,000 were functioning well at last follow up.

Additionally, our study has also found no association between donor LBTs and pancreas graft outcome. Hence, donor amylase and LBTs alone should not be a determining factor in organ utilisation in the modern era of pancreas transplantation.

With the rising demand for pancreas transplantation due to the increasing global disease burden of diabetes mellitus [1–3] and longer waiting lists there is a need to widen access to pancreas transplantation through improved utilisation of grafts. Further knowledge and evidence-based organ assessment is crucial in quantifying extended-criteria and marginal donor organs [21]. At the time of organ selection, some serological markers such as amylase levels and liver blood tests can be useful taken together with other markers of increasing risk when deciding the suitability and quality of a pancreas allograft but it is important to note they are non-specific and that there are other donor variables that may affect these blood tests [10–12, 22]. Nonetheless, surgeons remain reluctant to accept pancreas grafts from donor with raised serum amylase due to concerns of inferior outcomes. This becomes more important during an era of DCD transplantation as these are more prone to ischaemic damage but represent an underused resource [23–25].

Vinkers et al established the Pre-procurement Pancreas Allocation Suitability Score (P-PASS) in 2008, where a total of nine clinical parameters were used to predict the odds of a donor allograft being accepted for transplantation. The P-PASS score includes donor body mass index, age, duration of intensive care stay, serum amylase, lipase, sodium, duration of donor cardiac arrest, and whether or not the donor was on vasopressor support. Liver function tests, cold ischaemia time and type of donor, i.e., DCDs vs. DBDs are excluded in P-PASS. A low P-PASS score of 17 and below were three times more likely to be accepted as pancreas donors than donor grafts that scored above 17 [20]. The P-PASS score has been utilised by Eurotransplant since 2009 [26]. Amylase levels were among the nine parameters in this scoring system, where raised Amylase of ≥130 iu/L contributes to a higher P-PASS score, which is associated with high odds of organ discard [20]. It is important to note that the P-PASS score was developed based on chance of organ decline, and not based on outcome in transplanted pancreases. It therefore reflects what clinicians perceive as high risk, rather than factors which actually predict pancreas quality.

Interestingly, two retrospective analyses by Schenker et al and Blok JJ et al [27] revealed that there is no significant difference in long-term patient and graft survivals between donors with low (≤17) and high (≥17) P-PASS scores [28]. This supports our findings and further reiterates that donor pancreas allografts should not be rejected based solely on high P-PASS scores and the parameters that deem a subgroup of donors as marginal donors.

In the US the Pancreas Donor Risk index was developed from data taken from the Scientific Registry of Transplant Recipients database and is linked to graft survival. It has also been validated in the UK cohort [29]. It may offer better predictions for more marginal pancreases and some studies have confirmed it is a better predictor of pancreas graft survival after SPK rather than after solitary pancreas transplantation [30]. It is also a better predictor than the P-PASS for pancreas graft survival [27]. Age, and cold ischaemia are included but amylase and lipase are excluded from the PDRI, as they were not associated with outcome. A recent systematic review conducted by Ling et al have shown that both P-PASS and PDRI are inadequate risk indices for use in solid pancreas transplantation due inadequate reporting of model performance metrics outside of current externally validated cohorts. P-PASS was derived for pancreas graft acceptance and not for prediction of graft survival. PDRI was validated for the outcomes of 1-year pancreas survival, and limited to graft survival for SPK transplants only [31]. These studies also did not focus on donor blood tests, and their impact on outcome, our study fills these gaps.

Liver function tests and amylase are both included in the North American Islet donor score which was developed to guide decision making as to whether to accept a particular pancreas to improve isolation outcomes [32, 33]. However, both amylase and transaminases were shown in the same Wang 2016 paper to have no impact on success of islet isolation from 1,056 donors. This mirrors our results in whole pancreas transplantation.

Additionally, it is worth noting that a previous smaller study by Hesse and Sutherland have demonstrated that an isolated elevation of amylase is not usually related to the functional status of the pancreas allograft, unless there was overt pancreatic trauma or pancreatitis. Graft function post-transplantation was found to be comparable in the recipients, regardless of whether the donor had normal or elevated amylase levels [34]. Krieger and others further echoes this, as they have shown that SPK graft survival rates in recipients of grafts from donors who had raised serum amylase compared favourably to outcomes in recipients of “ideal” donor grafts [35].

There are some limitations to the studies discussed above; both were performed in the early phases of pancreas transplantation, and only confined to the United States. Furthermore, the sample sizes in both studies were smaller than the present study. Both studies also reviewed graft outcomes based on arbitrary categories of normal and abnormal serum amylase, which reduces the power of the study [15–18]. Despite the limitations, these studies support our findings that hyperamylaseamia in donors is not a contraindication for pancreas organ donation. To our knowledge, our work is the largest cohort study to date, looking at the relationship between serum amylase and liver function tests upon pancreas graft survival in the modern era of pancreas transplantation. We have incorporated prospectively collected data from a large cohort, with robust statistical analysis as detailed above.

With the increased use of DCD grafts, there is an increased vulnerability towards inevitable ischaemic-reperfusion injury during procurement [36, 37]. Due to the close anatomical relationship between the pancreas and its partially shared vascular supply with the foregut, raised donor LBTs may represent ischaemic injury to abdominal viscera [14, 38–42]. Raised liver blood tests (LBTs) in liver donors were frequently used to define extended-criteria donors, in the context of liver transplantation [43, 44]. Due to the partially shared vascular supply [14, 38–42] between liver and pancreas we hypothesised that elevations in LBTs, especially transaminases, reflect hypoxic injury to the liver and are therefore a surrogate for hypoxic injury to the pancreatic allograft. This is supported by work showing that donors dying from hypoxic brain injury have far higher transaminase levels [11, 12].

Parajuli and others have found that delayed kidney graft function represented a significant risk factor for early pancreas graft loss (<90 days post-transplant) in SPK transplant recipients [45]. In view of this, we have therefore separately assessed the impact of peak donor renal function tests in our study, as the function of the transplanted kidney can impact pancreas graft function [45, 46]. We have found that donor renal blood tests did not predict pancreas graft survival (Supplementary Table S3). However, transplanted kidney graft survival may be better assessed in a study dedicated to kidney grafts, with much larger cohorts of kidney transplants alone.

More recently, our group explored the significance of deranged LBTs in liver transplantation and found that raised donor transaminases do not predict post-liver transplant outcomes [13]. Our study mirrors these findings in pancreas transplant, as there were no associations between abnormal LBTs and pancreas graft survival. Since routine liver function tests are carried out as part of the work up for a potential transplant donor, our findings reinforces that rises in LBTs should not be considered as a limiting factor in pancreas allograft allocation.

Furthermore, whilst in intensive care units, some donors may be given insulin in response to donor hyperglycaemia of varying aetiologies [47, 48]. A recent, large cohort study by Shapey et al suggests that donor insulin use is associated with a higher risk of graft loss due to islet failure and a lower risk of graft loss due to thrombosis in pancreas transplant recipients [49]. This suggests that actual markers of organ function and pancreas physiology may be more predictive of pancreas transplant outcomes, rather than non-specific enzyme release, such as amylase.

This study is limited by the retrospective design. Specifically, we lack granularity of data regarding imaging and clinical features of acute pancreatitis, or details regarding pancreatic trauma. As we only included donated pancreas grafts which were accepted and used for transplantation, the vast majority will be from donors without clinical or radiological features of pancreatitis or pancreatic trauma. Therefore, we cannot comment on the suitability of pancreases from donors where these features are present. We also lack information on serum lipase. Though we acknowledge it is a more specific marker of pancreatic injury, it is not routinely performed in the UK setting. Further study into the effects of lipase and pancreas graft transplantation outcomes, in a healthcare system that routinely measures donor serum lipase, may be a point in future research.

There is also a degree of selection bias, as various clinicians have different thresholds for donor amylase when it comes to discarding grafts at the time of organ procurement. As described in our results section, there is a wide range of donor amylase values in the pancreas grafts that were transplanted in our study. Hence multivariable analysis was performed to adjust for key confounders.

Finally, the right skewed distribution of serum blood tests translates to smaller number of donors in the extremely elevated results. This is reflected in the marked increase in the confidence intervals of the cox regression model adjusted hazard ratios with restricted cubic splines (Figure 2). The low number of donor with high amylase may affect the power of our study, and the most powerful way of assessing this was by using restricted cubic splines (Figure 2). The confidence intervals around these splines reveal uncertainty as amylase level increases. These are confidence fairly narrow up to a peak amylase value of 500, and then sharply increase due to the lower numbers of pancreases transplanted from donors with amylase values greater than 500. Although pancreases from donors with severely increased peak amylase (>1,000) all performed well in this study, this is a small group. Therefore it remains uncertain whether large increases in amylase (>1,000) impact on graft survival, and such donors should be assessed, on a case-by-case basis.

In conclusion, our study has demonstrated that the use of pancreas grafts from donors with hyperamylasaemia and raised liver blood tests is not associated with inferior outcomes. Mild or moderately raised donor amylase and liver blood tests should therefore not be considered a barrier to transplantation and organ utilisation when other donor factors are considered acceptable. This knowledge should prevent unnecessary organ discard, and provides a simple method to expand the donor pool to meet current demands.

## Data Availability

The original contributions presented in the study are included in the article/Supplementary Material, further inquiries can be directed to the corresponding author.

## Abbreviations

ALT, alanine transaminase; AST, aspartate transaminase; ALP, alkaline phosphatase; CIT, Cold Ischaemic Time; DCD, Donor after Circulatory Death; DBD, Donor after Brainstem Death; DM, Diabetes Mellitus; LBT, Liver Blood Tests; NHS, National Health Service (United Kingdom); NHSBT, National Health Service Blood and Transplant; SPK, Simultaneous Pancreas-Kidney transplantation; WIT, Warm Ischaemic Time.
