# Supplementary material for: Donor Blood Tests do Not Predict Pancreas Graft Survival After Simultaneous Pancreas Kidney Transplantation; a National Cohort Study
Source: Transpl Int. 2024 May 20;37:12864. doi: 10.3389/ti.2024.12864 (PMC11144863; doi:10.3389/ti.2024.12864)
Supplement: Supplementary file 1 [file DataSheet2.docx]

Supplementary Tables

Supplementary Table 1: Full cohort demographics (N = 857), inclusive of missing data. DBD – donation following brainstem death; DCD – donation following circulatory death

|  | **DBD (N=619)** | **DCD**  **(N=238)** | **Overall (N=857)** |
| --- | --- | --- | --- |
| **Recipient Age** |  |  |  |
| Mean (SD) | 42.6 (8.77) | 41.2 (8.90) | 42.3 (8.82) |
| Median [Min, Max] | 42.0 [21.0, 64.0] | 42.0 [20.0, 61.0] | 42.0 [20.0, 64.0] |
| **Recipient Sex** |  |  |  |
| Female | 267 (43.1%) | 92 (38.7%) | 359 (41.9%) |
| Male | 352 (56.9%) | 146 (61.3%) | 498 (58.1%) |
| **Recipient Ethnicity** |  |  |  |
| White | 515 (83.2%) | 205 (86.1%) | 720 (84.0%) |
| Non-White | 97 (15.7%) | 32 (13.4%) | 129 (15.1%) |
| Missing | 7 (1.1%) | 1 (0.4%) | 8 (0.9%) |
| **Recipient BMI** |  |  |  |
| Mean (SD) | 25.0 (3.57) | 25.5 (3.66) | 25.1 (3.60) |
| Median [Min, Max] | 24.6 [17.7, 36.5] | 25.1 [18.4, 36.9] | 24.8 [17.7, 36.9] |
| Missing | 84 (13.6%) | 39 (16.4%) | 123 (14.4%) |
| **Recipient Dialysis** |  |  |  |
| Haemodialysis | 238 (38.4%) | 70 (29.4%) | 308 (35.9%) |
| Peritoneal Dialysis | 151 (24.4%) | 63 (26.5%) | 214 (25.0%) |
| Not on Dialysis | 230 (37.2%) | 105 (44.1%) | 335 (39.1%) |
| **Type of Recipient Diabetes** |  |  |  |
| Type 1 Diabetes Mellitus | 488 (78.8%) | 179 (75.2%) | 667 (77.8%) |
| Type 2 Diabetes Mellitus | 22 (3.6%) | 10 (4.2%) | 32 (3.7%) |
| Missing | 109 (17.6%) | 49 (20.6%) | 158 (18.4%) |
| **Transplant Year** |  |  |  |
| 2016 | 103 (16.6%) | 44 (18.5%) | 147 (17.2%) |
| 2017 | 124 (20.0%) | 48 (20.2%) | 172 (20.1%) |
| 2018 | 126 (20.4%) | 48 (20.2%) | 174 (20.3%) |
| 2019 | 112 (18.1%) | 45 (18.9%) | 157 (18.3%) |
| 2020 | 74 (12.0%) | 23 (9.7%) | 97 (11.3%) |
| 2021 | 80 (12.9%) | 30 (12.6%) | 110 (12.8%) |
| **Donor Sex** |  |  |  |
| Female | 316 (51.1%) | 96 (40.3%) | 412 (48.1%) |
| Male | 303 (49.0%) | 142 (59.7%) | 445 (51.9%) |
| **Donor Age** |  |  |  |
| Mean (SD) | 34.9 (13.5) | 31.0 (13.1) | 33.8 (13.5) |
| Median [Min, Max] | 35.0 [10.0, 63.0] | 29.0 [4.00, 54.0] | 34.0 [4.00, 63.0] |
| **Donor BMI** |  |  |  |
| Mean (SD) | 23.4 (3.31) | 22.6 (3.64) | 23.2 (3.43) |
| Median [Min, Max] | 23.4 [14.5, 38.4] | 22.6 [11.3, 36.2] | 23.1 [11.3, 38.4] |
| **Donor Ethnicity** |  |  |  |
| White | 554 (89.5%) | 216 (90.8%) | 770 (89.8%) |
| Non-White | 53 (8.6%) | 21 (8.8%) | 74 (8.6%) |
| Missing | 12 (1.9%) | 1 (0.4%) | 13 (1.5%) |
| **Donor Cause of Death** |  |  |  |
| Hypoxic Brain Injury | 198 (32.0%) | 111 (46.6%) | 309 (36.1%) |
| Intrcranial Haemorrhage | 284 (45.9%) | 61 (25.6%) | 345 (40.3%) |
| Intrcranial Thrombosis | 27 (4.4%) | 9 (3.8%) | 36 (4.2%) |
| Trauma | 30 (4.8%) | 25 (10.5%) | 55 (6.4%) |
| Other | 55 (8.9%) | 17 (7.1%) | 72 (8.4%) |
| Missing | 25 (4.0%) | 15 (6.3%) | 40 (4.7%) |
| **Cold Ischaemic Time (minutes)** |  |  |  |
| Mean (SD) | 653 (129) | 628 (122) | 646 (127) |
| Median [Min, Max] | 647 [223, 1320] | 611 [339, 1060] | 634 [223, 1320] |
| Missing | 21 (3.4%) | 10 (4.2%) | 31 (3.6%) |
| **Warm Ischaemic Time (minutes)** |  |  |  |
| Mean (SD) | 41.0 (59.8) | 39.0 (20.2) | 40.4 (52.0) |
| Median [Min, Max] | 34.0 [0, 759] | 36.0 [0, 253] | 34.0 [0, 759] |
| Missing | 44 (7.1%) | 20 (8.4%) | 64 (7.5%) |
| **Normothermic Regional Perfusion** |  |  |  |
| No | 619 (100%) | 211 (88.7%) | 830 (96.8%) |
| Yes | 0 (0%) | 27 (11.3%) | 27 (3.2%) |
| **1-year Graft failure** |  |  |  |
| No | 561 (90.6%) | 220 (92.4%) | 781 (91.1%) |
| Yes | 54 (8.7%) | 15 (6.3%) | 69 (8.1%) |
| Missing | 4 (0.6%) | 3 (1.3%) | 7 (0.8%) |
| **3-year Graft failure** |  |  |  |
| No | 547 (88.4%) | 214 (89.9%) | 761 (88.8%) |
| Yes | 68 (11.0%) | 21 (8.8%) | 89 (10.4%) |
| Missing | 4 (0.6%) | 3 (1.3%) | 7 (0.8%) |

Supplementary Table 2: Full summary of peak donor serum amylase, liver blood tests, renal function tests and serum lactate, inclusive of missing data. DBD – donation following brainstem death; DCD – donation following circulatory death; ALT – alanine transaminase; AST – aspartate transaminase; ALP – alkaline phosphatase; eGFR – estimated glomerular filtration rate.

|  | **DBD (N=619)** | | | | | **DCD (N=238)** | | | **Overall (N=857)** | |
| --- | --- | --- | --- | --- | --- | --- | --- | --- | --- | --- |
| **Amylase** |  | | | | |  | | |  | |
| Mean (SD) | 131 (212) | | | | | 108 (131) | | | 125 (194) | |
| Median [Min, Max] | 70.0 [8.00, 3300] | | | | | 69.0 [10.0, 1310] | | | 70.0 [8.00, 3300] | |
| Missing | 89 (14.4%) | | | | | 41 (17.2%) | | | 130 (15.2%) | |
| **ALT** |  | | | | |  | | |  | |
| Mean (SD) | 196 (434) | | | | | 201 (444) | | | 198 (437) | |
| Median [Min, Max] | 59.0 [8.00, 5090] | | | | | 88.5 [9.00, 5930] | | | 67.0 [8.00, 5930] | |
| Missing | 18 (2.9%) | | | | | 6 (2.5%) | | | 24 (2.8%) | |
| **AST** |  | | | | |  | | |  | |
| Mean (SD) | 133 (221) | | | | | 233 (824) | | | 159 (461) | |
| Median [Min, Max] | 65.0 [0, 2040] | | | | | 94.0 [10.0, 7910] | | | 72.0 [0, 7910] | |
| Missing | 352 (56.9%) | | | | | 145 (60.9%) | | | 497 (58.0%) | |
| **ALP** |  | | | | |  | | |  | |
| Mean (SD) | 99.5 (58.7) | | | | | 116 (74.9) | | | 104 (64.0) | |
| Median [Min, Max] | 85.0 [31.0, 721] | | | | | 90.0 [35.0, 541] | | | 86.0 [31.0, 721] | |
| Missing | 2 (0.3%) | | | | | 2 (0.8%) | | | 4 (0.5%) | |
| **Bilirubin** |  | | | | |  | | |  | |
| Mean (SD) | 14.4 (10.7) | | | | | 14.3 (9.72) | | | 14.4 (10.4) | |
| Median [Min, Max] | 12.0 [3.00, 124] | | | | | 11.5 [3.00, 65.0] | | | 12.0 [3.00, 124] | |
| Missing | 1 (0.2%) | | | | | 0 (0%) | | | 1 (0.1%) | |
| **Urea** | | |  | |  | | |  | |  |
| Mean (SD) | | | 7.76 (6.96) | | 8.11 (7.76) | | | 7.86 (7.19) | |  |
| Median [Min, Max] | | | 6.20 [2.20, 78.0] | | 6.60 [2.10, 86.0] | | | 6.30 [2.10, 86.0] | |  |
| **Creatinine** | | |  | |  | | |  | |  |
| Mean (SD) | | | 98.2 (52.7) | | 89.9 (41.6) | | | 95.9 (50.0) | |  |
| Median [Min, Max] | | | 87.0 [4.00, 604] | | 84.0 [20.0, 356] | | | 86.0 [4.00, 604] | |  |
| **eGFR** | | |  | |  | | |  | |  |
| Mean (SD) | | | 82.3 (16.3) | | 85.6 (17.9) | | | 83.1 (16.7) | |  |
| Median [Min, Max] | | | 90.0 [39.0, 231] | | 90.0 [21.0, 184] | | | 90.0 [21.0, 231] | |  |
| Missing | | 109 (17.6%) | | 72 (30.3%) | | | 181 (21.1%) | | |  |
| **Lactate** | |  | |  | | |  | | |  |
| Mean (SD) | | 2.20 (1.68) | | 1.97 (1.39) | | | 2.13 (1.61) | | |  |
| Median [Min, Max] | | 1.70 [0.400, 14.2] | | 1.50 [0.500, 11.7] | | | 1.70 [0.400, 14.2] | | |  |
| Missing | | 8 (1.3%) | | 7 (2.9%) | | | 15 (1.8%) | | |  |

Supplementary Table 3: 3-Year Graft Survival Cox regression using pooled data on peak values of donor blood tests from imputed datasets, inclusive of renal function tests and donor serum lactate.

*For blood tests, logs were taken before inclusion in this model, due to all blood tests results being right-skewed. The effect estimates relate to a unit increase in log2(blood tests value). Results from the various LBTs (ALT, AST, ALP, and bilirubin), renal function tests (urea, creatinine and eGFR) and serum lactate could not be included in a single model because of multicollinearity; therefore, multivariable results for each LBT are from a separate multivariable model. Multivariable results for variables other than LBTs are from the model including peak Amylase.*

ALT, alanine transaminase; AST, aspartate transaminase; ALP, alkaline phosphatase; CI, confidence interval; eGFR, estimated glomerular filteration rate; LBT, liver blood test; HR, hazard ratio; DBD, donation following brainstem death; DCD, donation following circulatory death

|  | **Adjusted HR (95% CI)** | **P-value** |
| --- | --- | --- |
| **Blood Tests** |  |  |
| **Urea (Peak)** | 1.026 (0.731 – 1.441) | 0.881 |
| **Creatinine (Peak)** | 1.043 (0.719 – 1.514) | 0.823 |
| **eGFR (Peak)** | 1.290 (0.482 – 3.450) | 0.601 |
| **Lacate (Peak)** | 0.769 (0.501 – 1.181) | 0.230 |
|  |  |  |
| **Amylase (Peak)** | 0.944 (0.754 – 1.181) | 0.602 |
| **ALT (Peak)** | 0.967 (0.848 – 1.102) | 0.616 |
| **AST (Peak)** | 0.908 (0.771 – 1.070) | 0.247 |
| **ALP (Peak)** | 0.865 (0.594 – 1.261) | 0.451 |
| **Bilirubin (Peak)** | 1.229 (0.930 – 1.624) | 0.148 |
|  |  |  |
| **Cold Ischaemic Time (hours)** | 1.338 (0.611 – 2.930) | 0.467 |
|  |  |  |
| **Donor Age (years)** | 1.009 (0.992 – 1.026) | 0.322 |
| **Donor Type** | 0.731 (0.430 – 1.243) | 0.247 |
| **Donor BMI** | 1.078 (1.015 – 1.144) | 0.014 |
|  |  |  |
| **Transplant Year** | 0.948 (0.820 – 1.096) | 0.472 |
|  |  |  |
| **Recipient Age (years)** | 0.960 (0.935 – 0.986) | 0.003 |
| **Recipient BMI** | 0.992 (0.918 – 1.073) | 0.842 |
