# Supplementary material for: Donor Blood Tests do Not Predict Pancreas Graft Survival After Simultaneous Pancreas Kidney Transplantation; a National Cohort Study
Source: Transpl Int. 2024 May 20;37:12864. doi: 10.3389/ti.2024.12864 (PMC11144863; doi:10.3389/ti.2024.12864)

Supplementary Figure

Supplementary Figure 1: Donor peak aspartate transaminase (AST), peak alkaline phosphatase (ALP) and peak bilirubin distribution. 1A and 1B shows values of peak AST across the entire cohort displayed in histogram and violin plot respectively. 1C and 1D shows values of peak ALP across the entire cohort displayed in histogram and violin plot respectively. 1E and 1F shows values of peak bilirubin across the entire cohort displayed in histogram and violin plot respectively.


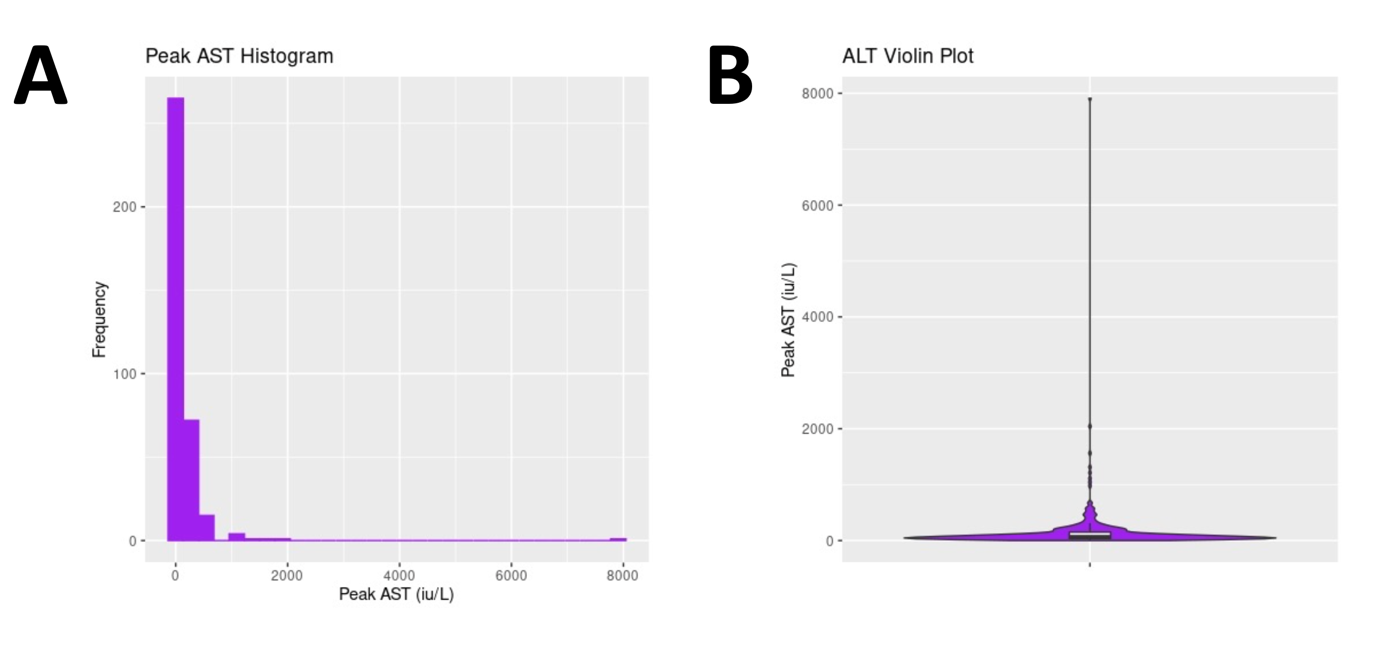

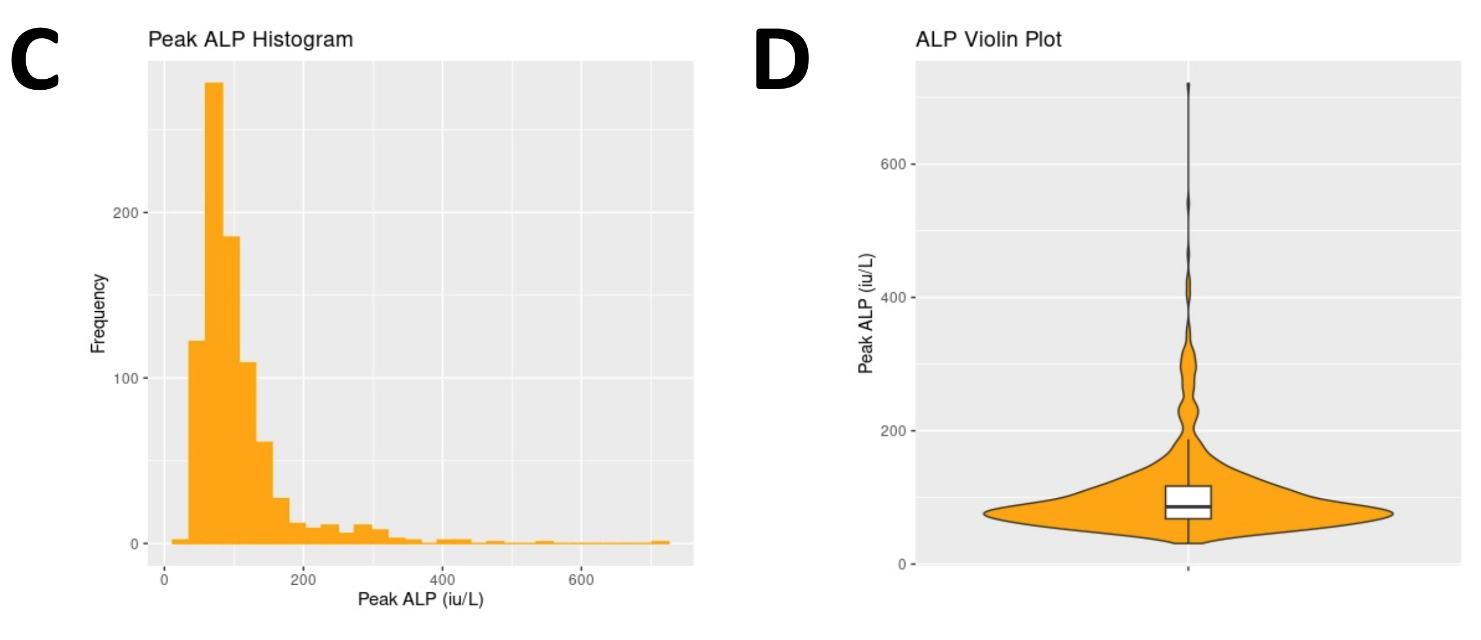

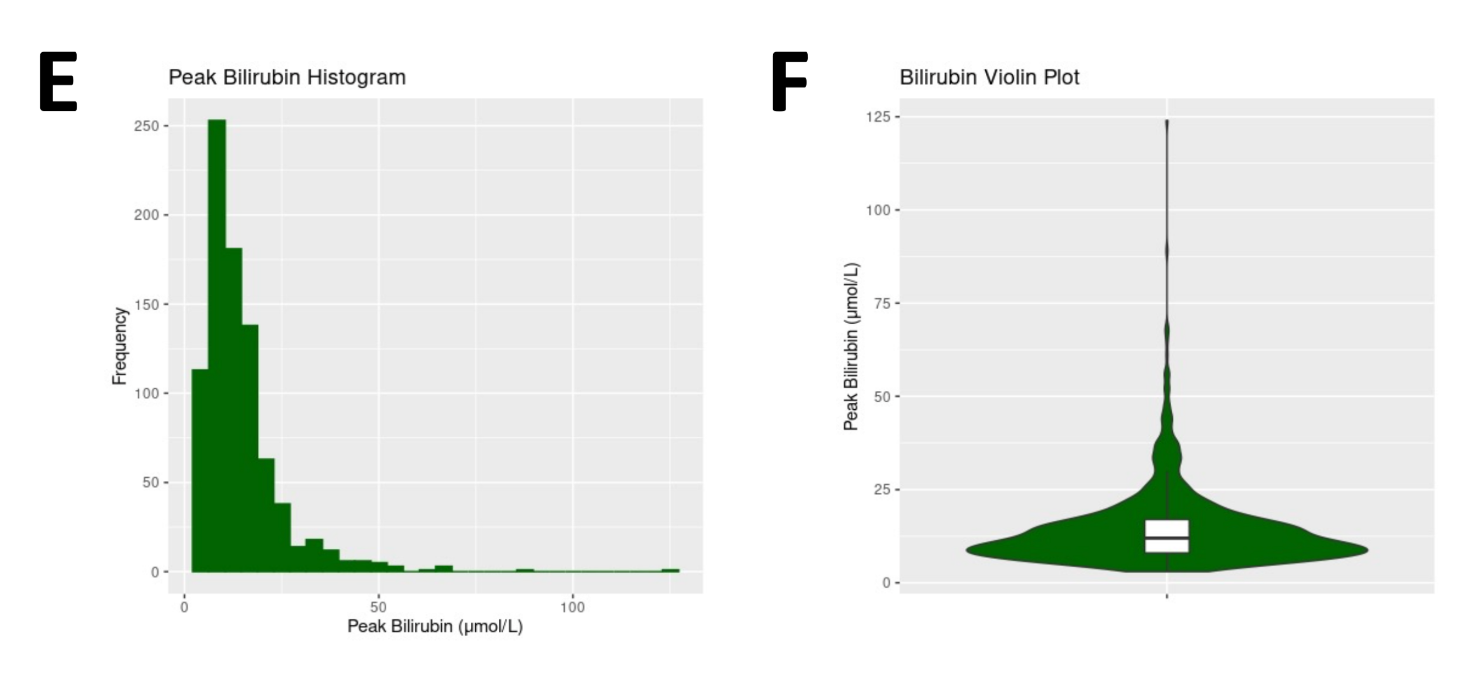

Supplement: Supplementary file 2 [file DataSheet1.docx]
